# Ultrasound-guided central venous catheter placement: first things first

**DOI:** 10.1186/s13054-017-1921-9

**Published:** 2017-12-29

**Authors:** Bernd Saugel, Leonie Schulte-Uentrop, Thomas W. L. Scheeren, Jean-Louis Teboul

**Affiliations:** 1 Department of Anesthesiology, Center of Anesthesiology and Intensive Care Medicine, University Medical Center Hamburg-Eppendorf, Martinistrasse 52, 20246 Hamburg, Germany; 2 Department of Anesthesiology, University of Groningen, University Medical Centre Groningen, Groningen, The Netherlands; 3 Service de Réanimation Médicale Hôpital de Bicêtre, Hôpitaux Universitaires Paris-Sud, AP-HP, Le Kremlin-Bicêtre, France

We thank Drs. Gawda and Czarnik for their comments [1] regarding our article on ultrasound (US)-guided central venous catheter (CVC) placement [2].

We recommended a “six-step-approach”. Because US is still used infrequently for CVC insertion [3, 4] we aimed to provide a basic, pragmatic, and evidence-based concept applicable in clinical routine rather than making the approach unnecessarily complicated.

We discussed that a disadvantage of the short-axis/out-of-plane approach is that not the entire needle but only an echogenic point (that must not be the needle tip) is visualized [2].

Experienced users might perform additional steps during US-guided CVC placement, e.g., double-checking that the needle is not localized over the adjacent artery before entering the target vein (long-axis/in-plane approach). Although this additional step might further improve procedural quality in the hands of experts, it is not backed up by study data and necessitates transiently abandoning the view of the needle and the target vein. This “angle change” of the US probe to check the adjacent artery before puncturing the vein only makes sense if the artery is anatomically located equally deep or deeper (and not more superficial) compared to the vein.

US can provide important information about the correct position of the guidewire [2]. As suggested, one can check the ipsilateral subclavian vein (for internal jugular vein CVCs) or the internal jugular vein (for subclavian CVCs) to exclude misdirection of the guidewire. Consequently, following this line of argument, one should also exclude misdirection of the guidewire in the contralateral veins and the thyroid veins. It needs to be emphasized, however, that strictly aseptic conditions need to be ensured during all additional steps; this might even require a second operator excluding misdirection of the guidewire in different veins with an additional non-sterile US probe. Alternatively, one could confirm the tip of the guidewire in the superior vena cava/the right atrium using transthoracic echocardiography.

Although not part of our basic recommendations, experienced operators can use US to confirm the correct position of the CVC (direct visualization of the CVC tip or indirect identification of the CVC tip by rapid injection of saline and imaging of turbulent flow in the right atrium or vascular structures) and to exclude iatrogenic pneumothorax [5].

US offers various opportunities to improve the quality and safety of CVC placement, but to advocate and promote US as the standard of care for CVC placement in anesthesiology and critical care our recommendations need to be straightforward!

## Abbreviations

CVC: Central venous catheter
US: Ultrasound
